# Association of Autoantibodies to BP180 with Disease Activity in Greek Patients with Bullous Pemphigoid

**DOI:** 10.1155/2012/854795

**Published:** 2012-11-27

**Authors:** Aikaterini Patsatsi, Aikaterini Kyriakou, Aikaterini Pavlitou-Tsiontsi, Anastasia Giannakou, Dimitrios Sotiriadis

**Affiliations:** ^1^2nd Department of Dermatology and Venereology, Papageorgiou General Hospital, Aristotle University School of Medicine, Nea Eflkarpia, Ring Road Thessalonikis, 56403 Thessaloniki, Greece; ^2^Immunology Laboratory, Papageorgiou General Hospital, Nea Eflkarpia, Ring Road Thessalonikis, 56403 Thessaloniki, Greece

## Abstract

39 bullous pemphigoid (BP) patients were studied to assess the clinical significance of anti-BP180 and anti-BP230 circulating autoantibodies of BP and correlate their titers with the clinical scores of the BP Disease Area Index (BPDAI) and the Autoimmune Bullous Skin Disorder Intensity Score (ABSIS) as well as with the intensity of pruritus measured by the BPDAI pruritus component. All parameters were evaluated by the time of diagnosis (baseline), month 3, and month 6. Titers of anti-BP180 autoantibodies were strongly correlated with BPDAI (*r* = 0.557, *P*  value < 0.0001) and ABSIS (*r* = 0.570, *P*  value < 0.0001) values, as well as with BPDAI component for the intensity of pruritus (rho = 0.530, *P*  value = 0.001) at baseline. At month 3, titers of anti-BP180 autoantibodies were strongly correlated with BPDAI (rho = 0.626, *P*  value = 0.000) and ABSIS (rho = 0.625, *P*  value = 0.000) values, as well as with the BPDAI component for the intensity of pruritus (rho = 0.625, *P*  value = 0.000). At month 6, titers of anti-BP180 autoantibodies were strongly correlated with BPDAI (rho = 0.527, *P*  value = 0.001) and ABSIS (rho = 0.526, *P*  value = 0.001) values, as well as with the BPDAI component for the intensity of pruritus (rho = 0.525, *P*  value = 0.001). There was no statistically significant correlation between titers of anti-BP230 autoantibodies and the BPDAI, ABSIS, and BPDAI component for the intensity of pruritus at the same time points.

## 1. Introduction

Bullous pemphigoid (BP) is the most common subepidermal autoimmune bullous disease. Detection of anti-BP180 and/or anti-BP230 serum autoantibodies by commercially available ELISA kits has almost become nowadays a routine method for the diagnosis and followup of BP patients [1–3].

During the last decade, efforts to evaluate the clinical extent and severity of autoimmune bullous diseases, generally, have led to the establishment of scoring systems. Up to today there are two already validated scoring systems for pemphigus, the Autoimmune Bullous Skin Disorder Intensity Score (ABSIS) and the Pemphigus Disease Area Index (PDAI) [4].

ABSIS has been introduced in 2007, in order to achieve an improved evaluation and monitoring of the status of both oral and cutaneous lesions in patients with pemphigus. It has been used, though, in other autoimmune bullous diseases, such as BP and epidermolysis bullosa acquisita [5]. The Pemphigus Disease Area Index (PDAI) was developed by the International Pemphigus Definitions Committee in 2008, to evaluate both mucosal and cutaneous lesions [6]. 

In 2011, the International Pemphigoid Committee proposed the BP Disease Area Index (BPDAI), in order to achieve a consistent reporting of the outcomes in BP reports and studies [7]. BPDAI has small differences from the PDAI scoring sheet, as there is more emphasis given to the lesions on the extremities, than face, scalp, and mucosa. There are separate columns for the extent of blistering and for the urticarial/eczematous lesions that may be more extensive in BP [7].

In the present study, we correlated two clinical scores, not yet validated for bullous pemphigoid (BPDAI, ABSIS), and the BPDAI component for the intensity of pruritus, with the titers of circulating autoantibodies (anti-BP180, anti-BP230), in a group of Greek BP patients, in order to assess the disease activity during the first six months after diagnosis.

## 2. Materials and Methods

39 patients suffering from BP were consecutively selected to participate in this prospective cohort study. Diagnosis of BP was confirmed, before the initiation of any treatment, by histology, direct immunofluorescence, and detection of circulating anti-BP180 and anti-BP230 autoantibodies by ELISA (commercially available MBL kits, Japan). The cut-off value for the MBL assays was that of 9.0 U/mL and the upper detection range was 150.0 U/mL. 

Titers of circulating autoantibodies were measured by the time of diagnosis (baseline) and every three months thereafter until month 6 (month 3, month 6). At the same time points (baseline, month 3, and month 6), the clinical severity was evaluated using the BPDAI and ABSIS scores, as well as the BPDAI component for the intensity of pruritus. BPDAI has a score of 0–120 for three distinct parameters (total 360): number and size of bullous lesions, number and size of erythematous nonbullous lesions, and number and size of mucosal lesions. In our group of patients, there were no mucosal lesions. ABSIS has a range from 0 to 206. It measures the extent and quality of skin lesions (0–150), the extent (0–11) and severity of mucosal lesions (0–45). BPDAI pruritus component ranges from 0 to 30. It measures the severity of itch during the past 24 hours (0–10), the past week (0–10), and the past month (0–10).

Administration of systemic prednisolone at a dose of 0.5 mg per kg BW per day and tapering upon response was the induction therapeutic scheme, which was applied to all patients. 

The ethics board approval and patients' informed consent were provided.

We did not include an age- and gender-matched control group in our study as the diagnostic ELISA procedures (anti-BP180 and anti BP 230) are well established and the specificity and sensitivity is proven up to now in many studies. We aimed to focus on the assessment of disease activity and on the use of the newly introduced clinical scoring systems.

### 2.1. Statistical Analysis

Descriptive statistics including the mean, the standard deviation (SD), the median, the minimum, and the maximum values were used in order to present continuous variables, while frequency distributions and percentages were used for categorical data. The normality of the continuous variables was tested with the Shapiro-Wilk test. Pearson's correlation coefficient and Spearman's rank test were also used to explore relationships between continuous variables. Preliminary analyses were performed to ensure no violation of the assumptions of normality, linearity, and homoscedasticity. Wilcoxon's signed rank test was performed to evaluate significant differences in the BPDAI, ABSIS, BPDAI components for the intensity of pruritus, anti-BP180 autoantibodies, and anti-BP230 autoantibodies between baseline and month 3, as well as between month 3 and month 6. All tests were two sided, and the significance level was chosen to be *α* = 0.05. Data analysis was performed using the statistical package for social sciences SPSS 15.0.

## 3. Results and Discussion

In total, 39 patients participated in the study. All patients remained under followup throughout the 6-month period. There was a female preponderance in our sample with 56.4% (*n* = 22) female patients and 43.6% (*n* = 17) male patients. The median patients' age was 76.0 (range: 28.0–91.0). Patients' clinical characteristics at baseline, month 3, and month 6 are summarized in Table 1. The number of patients with clinically active BP, positive anti BP 180 autoantibodies (≥9.0 U/mL), and positive anti BP 230 autoantibodies (≥9.0 U/mL) at all different time points are presented in Table 2.

In our BP patients during the first 6 months after diagnosis, there was a small number of recurrences (Table 2). After management of the extended eruption, most patients remained clear of lesions during the next follow-up visits, being under a maintenance dose of 5 mg prednisolone. Only 6 and 4 patients presented with a relapse of the disease at month 3 and 6, respectively (Table 3).

At baseline, a large, positive, statistically significant correlation was detected between anti-BP180 autoantibodies and (a) BPDAI (*r* = 0.557, *P* value < 0.0001), (b) ABSIS (*r* = 0.570, *P* value < 0.0001), and (c) BPDAI component for the intensity of pruritus (rho = 0.530, *P* value = 0.001). Therefore, high levels of anti-BP180 autoantibodies are associated with higher BPDAI, ABSIS, and BPDAI component for the intensity of pruritus. On the contrary, there was no statistically significant correlation between anti-BP230 autoantibodies and (a) BPDAI (rho = 0.206, *P* value = 0.208), (b) ABSIS (rho = 0.245, *P* value = 0 anti-BP180), and (c) BPDAI component for the intensity of pruritus (rho = 0.192, *P* value = 0.242), suggesting that high levels of anti-BP230 autoantibodies are not associated with higher BPDAI, ABSIS, and BPDAI component for the intensity of pruritus. 

At month 3, a large, positive, statistically significant correlation was detected between anti-BP180 autoantibodies and (a) BPDAI (rho = 0.626, *P* value = 0.000), (b) ABSIS (rho = 0.625, *P* value = 0.000), and (c) BPDAI component for the intensity of pruritus (rho = 0.625, *P* value = 0.000), whereas there was no statistically significant correlation between anti-BP230 autoantibodies and (a) BPDAI (rho = 0.135, *P* value = 0.411), (b) ABSIS (rho = 0.129, *P* value = 0.434), and (c) BPDAI component for the intensity of pruritus (rho = 0.105, *P* value = 0.525). 

At month 6, a large, positive, statistically significant correlation was detected between anti-BP180 autoantibodies and (a) BPDAI (rho = 0.527, *P* value = 0.001), (b) ABSIS (rho = 0.526, *P* value = 0.001), and (c) BPDAI component for the intensity of pruritus (rho = 0.525, *P* value = 0.001). On the other hand, no statistically significant correlation was detected between anti-BP230 autoantibodies and (a) BPDAI (rho = 0.308, *P* value = 0.057), (b) ABSIS (rho = 0.307, *P* value = 0.057), and (c) BPDAI component for the intensity of pruritus (rho = 0.313, *P* value = 0.052). 

The NC16A domain of BP180, located at the extracellular portion of BP180 or BPAg2, is the main targeted antigen in most cases of BP [1–3]. BP230 is an intracytoplasmic protein, and as autoantibodies are not considered to access into the intact keratinocytes, production of anti-BP230 autoantibodies may represent an epiphenomenon. To our knowledge, there is no study indicating the correlation between the titers of anti-BP180 and anti-BP230 autoantibodies with each other. The role of BP230 in BP pathogenesis remains unclear [2].

In many patients there are anti-BP230 autoantibodies by the time of diagnosis, and in some they are detected later in the course of the disease. Several studies suggest that anti-BP230 autoantibodies may play an important role in the onset of clinical symptoms and the formation of blisters [8], while others suggest that anti-BP230 autoantibodies may bind to the target antigen in injured keratinocytes or even in intact ones by penetrating the cells and possibly contribute to the perpetuation of disease [9]. 

There is a limited number of BP patients in which there are only anti-BP230 circulating autoantibodies, and thus, it is suggested to perform the detection of both, anti-BP180 and anti-BP230 autoantibodies, in all cases [1, 10, 11].

Still, according to a most recent study, there is about 8% of BP patients whose sera react to regions of BP180 exclusively outside of the NC16A domain, and thus, BP may not be identified using the currently available BP180 ELISA. Use of the BP230 ELISA in the above study would only have detected 1 of the 4 patients with non-NC16A BP [12].

In a number of studies, the diagnostic sensitivity and specificity of the commercially available assays has been elucidated, and moreover, it has been associated with the course of disease [13]. Titers of anti-BP180 autoantibodies have been reported to correlate with disease activity, pruritus severity, peripheral blood eosinophil counts, and disease duration [13–17].

Titers of anti-BP230 autoantibodies have been repeatedly reported not to parallel with the course and severity of BP [10, 13–18]. 

In accordance with the published experience, in our group of patients, titers of anti-BP180 autoantibodies were strongly correlated with the clinical scores BPDAI and ABSIS, as well as with the BPDAI component for the intensity of pruritus in three consecutive measurements, by the time of diagnosis (baseline), at month 3, and at month 6. On the other hand, there was no statistically significant correlation between titers of anti-BP230 autoantibodies and BPDAI, ABSIS, and BPDAI component for the intensity of pruritus. An additional observation was that the rate of reduction of anti-BP230 autoantibodies titers, in our small sample of BP patients, seemed to be slower from this of anti-BP180 autoantibodies titers after initiation of therapy.

## 4. Conclusions

Detection of both anti-BP180 and anti-BP230 autoantibodies is needed for the establishment of diagnosis in all cases of suspected BP. Followup of only anti-BP180 titers correlates with disease activity. Our first experience of the combined use of detection of circulating BP autoantibodies every three months with the completion of BPDAI and ABSIS scoring sheets was positive. We strongly believe that this combination offers an easy option for close followup of patients with BP. 

## Figures and Tables

**Table 1:** Patients' clinical characteristics at baseline, month 3, and month 6.

Clinical characteristics	Statistics	Values (*n* = 39) at baseline	Values (*n* = 39) at month 3			Values (*n* = 39) at month 6
	*P* value		0.000*		0.838	
BPDAI (0–360)	Mean ± SD	52.5 ± 24.7^#^		1.8 ± 4.5		1.5 ± 4.6
	Median (min–max)	50.0 (2.0–104.0)		0.0 (0.0–15.5)		0.0 (0.0–17.1)

	*P* value		0.000*		0.646	
ABSIS (0–206)	Mean ± SD	44.7 ± 21.7^#^		1.7 ± 4.1		1.4 ± 4.3
	Median (min–max)	43.5 (1.5–92.5)		0.0 (0.0–14.1)		0.0 (0.0–15.7)

	*P* value		0.000*		0.682	
BPDAI pruritus index (0–30)	Mean ± SD	23.1 ± 7.3		0.5 ± 1.3		0.5 ± 1.7
	Median (min–max)	24.0 (0.0–30.0)		0.0 (0.0–5.0)		0.0 (0.0–7.0)

	*P* value		0.000*		0.001*	
BP180 (U/mL)	Mean ± SD	87.9 ± 46.6^#^		11.1 ± 12.1		8.7 ± 9.7
	Median (min–max)	88.4 (10.0–190.8)		7.2 (2.3–49.1)		6.6 (1.9–41.1)

	*P* value		0.000*		0.115	
BP230 (U/mL)	Mean ± SD	36.5 ± 36.5		18.2 ± 12.9		15.7 ± 13.8
	Median (min–max)	26.4 (1.5–151.2)		16.9 (1.4–56.2)		9.0 (2.1–59.2)

*Wilcoxon's signed rank test statistically significant; ^#^variable normally distributed.

**Table 2:** Number of patients presenting clinically active BP, positive anti BP180 autoantibodies, and positive anti BP230 autoantibodies at all different time points.

	Baseline	Month 3	Month 6
Clinically active BP	39	6	4
Anti BP180 autoantibodies	39	7*	4
Anti BP230 autoantibodies	26	26	21

*One case had no signs of clinically active BP, but his anti BP180 autoantibodies had the borderline value of 9.2.

**Table 3:** BPDAI score, anti BP180 autoantibody titres, and anti BP230 autoantibody titres in patients who revealed a clinical relapse at different time points.

Case	Baseline			Month 3			Month 6		
	BPDAI	BP180	BP230	BPDAI	BP180	BP230	BPDAI	BP180	BP230
1	2.0	27.0	5.0	0.0	5.3	4.6	14.4	36.1	21.0
2	36.0	141.4	5.5	6.0	35.6	6.1	0.0	6.9	7.1
3	76.0	77.2	11.0	10.9	32.1	13.0	0.0	6.9	9.0
4	2.0	10.6	6.1	0.0	2.4	6.0	16.1	38.9	15.1
5	84.0	100.8	19.6	13.3	34.9	20.1	0.0	4.4	8.9
6	2.0	10.0	3.6	0.0	3.1	2.9	17.1	41.1	17.7
7	50.0	98.2	34.4	15.5	36.7	32.2	0.0	8.6	13.2
8	56.0	120.3	37.9	0.0	8.8	16.9	12.2	29.9	25.9
9	64.0	93.2	31.7	11.2	41.9	30.3	0.0	7.7	24.1
10	82.0	118.2	17.8	14.4	49.1	21.2	0.0	6.7	15.6
